# The Other Side of the Fascia: The Smooth Muscle Part 1

**DOI:** 10.7759/cureus.4651

**Published:** 2019-05-13

**Authors:** Bruno Bordoni, Marta Simonelli, Bruno Morabito

**Affiliations:** 1 Cardiology, Foundation Don Carlo Gnocchi, Milan, ITA; 2 Osteopathy, French-Italian School of Osteopathy, Pisa, ITA; 3 Osteopathy, School of Osteopathic Centre for Research and Studies, Milan, ITA

**Keywords:** fascia, myofascial, smooth muscle, osteopathic, mesoderm, fascia

## Abstract

According to current scientific standards, the fascia is a connective tissue derived from two separate germ layers, the mesoderm (trunk and limbs, part of the neck) and the ectoderm (cervical tract and skull). The fascia has the property of maintaining the shape and function of its anatomical district, but it also can adapt to mechanical-metabolic stimuli. Smooth muscle and non-voluntary striated musculature originated from the mesoderm have never been properly considered as a type of fascia. They are some of the viscera present in the mediastinum, in the abdomen and in the pelvic floor. This text represents the first article in the international scientific field that discusses the inclusion of some viscera in the context of what is considered fascia, thanks to the efforts of our committee for the definition and nomenclature of the fascial tissue of the Foundation of Osteopathic Research and Clinical Endorsement (FORCE).

## Introduction and background

Our Foundation of Osteopathic Research and Clinical Endorsement (FORCE) in our previous work defined the fascia as follows: “The fascia is any tissue that contains features capable of responding to mechanical stimuli. The fascial continuum is the result of the evolution of the perfect synergy among different tissues, liquids and solids, capable of supporting, dividing, penetrating, feeding and connecting all the districts of the body, from the epidermis to the bone, involving all the functions and organic structures. The continuum constantly transmits and receives mechano-metabolic information that can influence the shape and function of the entire body. These afferent/efferent impulses come from the fascia and the tissues that are not considered as part of the fascia in a bi-univocal mode [1].” This definition expands the definition of the Fascia Nomenclature Committee (2014), about what the fascia is and what it should include: “The fascial system includes adipose tissue, adventitia, neurovascular sheaths, aponeuroses, deep and superficial fasciae, dermis, epineurium, joint capsules, ligaments, membranes, meninges, myofascial expansions, periosteum, retinacula, septa, tendons (including endotendon/peritendon/epitendon/paratendon), visceral fasciae, and all the intramuscular and intermuscular connective tissues, including endomysium/perimysium/epimysium [2].” According to current scientific standards, the fascia is a connective tissue derived from two separate germ layers, the mesoderm and the ectoderm (cervical tract and skull) [3-6]. Fascia is the epidermis, the dermis, the adipose tissue, the skeletal muscle (with its connective tissue) and its tendons and ligaments, the circulatory and lymphatic system (vessels; blood and lymph), the meningeal tissue and nervous tissue, the joint capsule and bone tissue. The fascia has the property of maintaining the shape and function of its anatomical district, but it also can adapt to mechanical-metabolic stimuli, feeding the tissue [3-6]. Tissues consist of smooth muscle and non-voluntary striated musculature, originated from the mesoderm and with the properties described, they have never been properly considered. They are some of the viscera present in the mediastinum, in the abdomen and in the pelvic floor. The article highlights the presence of smooth and visceral striated muscle cells in different organs, underlying the mesodermal origin. Our committee of the Foundation of Osteopathic Research and Clinical Endorsement (FORCE), for the definition and nomenclature of the fascial tissue, discusses in this first article the inclusion of certain other viscera in the fascial field. This text continues with another article about heart embryological development, as well as muscles and collagen, concluding a scientific path to improve the definition of fascia.

## Review

Gastrointestinal tract mesodermal development

The mesodermal cells are organized into three sheets: paraxial mesoderm; intermediate mesoderm; lateral plate mesoderm. Paraxial mesoderm gives rise to somites, blocks of tissue running along both sides of the neural tube, which form muscle and the tissues of the back, the thorax and part of the neck, including connective tissue and the dermis. The gonads, kidney and reproductive tract are derived from intermediate mesoderm. The lateral plate mesoderm splits into parietal (somatic) and visceral (splanchnic) layers: the parietal layer forms the lateral body wall folds and the skeletal striated muscle, and the visceral mesoderm forms the walls of the gut tube [7]. The gastrointestinal tract forms from the endoderm (which gives rise to the epithelium) and the mesoderm. The mesoderm provides the form and contributes to the function of the apparatus, through smooth muscles, visceral striated muscles and other cells (mesenchymal cells, intestinal subepithelial myofibroblast). During gastrulation the wall of the gut is formed from the endoderm and the lateral plate mesoderm; the latter, with its inner portion, will surround the gut and will become the visceral mesoderm. At the same time, the neural crests will form the enteric system of the gastrointestinal complex. The mesoderm is essential for normal gut development (morphogenesis), because it transmits biochemical signals to the endoderm (for example fibroblast growth factor-4, bone morphogenetic proteins-4, forkhead box protein-1, small mother against decapentaplegic), which responds to the mesoderm through the Hedgehog (Hh) family; this type of signalling molecules transmits ontogenetic needs also to the endoderm. During development, these molecular interactions create the foregut (oesophagus, stomach, duodenum, liver, bile duct, pancreas, lungs and thyroid), the midgut (from which the portion of the jejunum, ileum, cecum and the ascending colon derive), and the hindgut (from which the transverse, descending and sigmoid colon and the rectum derive). This development begins in humans towards the end of the first quarter. The adult gastrointestinal system has very similar histology and can be divided into four concentric layers: mucosa, submucosa, muscle, and serious. The mucous membrane is the innermost layer, in contact with food. Its main functions are absorption, secretion and important digestion processes. It consists of an inner layer (epithelium) essential for the digestive processes, a basement membrane originated from the extracellular matrix, a lamina propria consisting of connective tissue and derived from the mesoderm, and a muscularis mucosae composed of several thin layers of smooth muscle fibres oriented in different ways (thanks to the mesoderm), propagating peristaltic waves. The submucosa (mesoderm) is a thin layer of connective tissue containing blood and lymphatic vessels and nerves that branch into the mucosa and in the muscularis externa. The muscular layer (mesoderm) is composed of smooth muscle fibres divided into an internal circular layer and an external longitudinal layer. Their coordinated contraction allows the bolus advancement, while the simultaneous contraction allows to mix food. The innermost layer has the function of preventing reflux. The serous membrane or adventitia (mesoderm) is the outermost connective tissue layer, that allows the passage of vessels and nerves. When the intestine is exposed in the abdominal cavity, the adventitia is called serous membrane (visceral peritoneum) and consists of a single layer of avascular flat nucleated cells, a simple squamous epithelium called mesothelium. When the bowel adheres to the walls of the abdominal cavity, the adventitia merges with the retroperitoneal tissues. The enteric plexus is present between the two layers, with the function of managing peristalsis. The epithelium is moved by mesenchymal-mesodermal cells, the intestinal subepithelial myofibroblast (ISEMFs); these are characterized by alpha-smooth muscle actin. The smooth muscles that give shape and function to the gastrointestinal tract are characterized by alpha and gamma-smooth muscle actin [8].

Cell, smooth muscle

**Figure 1:**
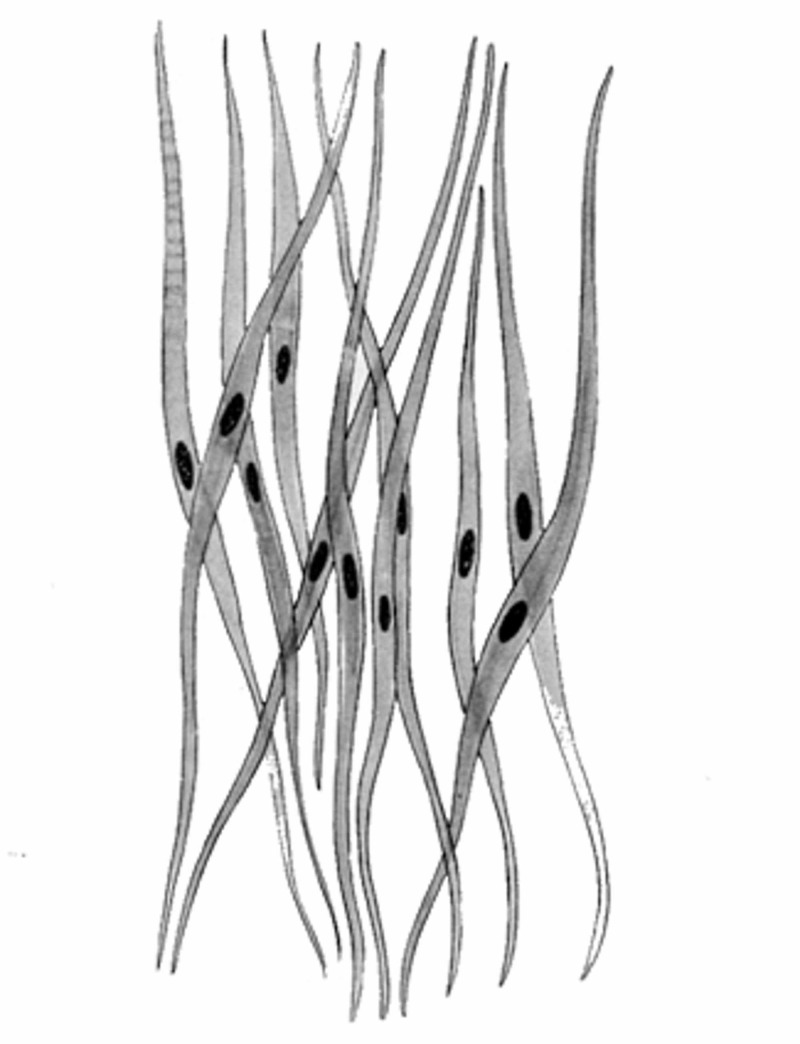
The image shows a bundle of fusiform smooth muscle cells. They are thickened in the middle area, where the nucleus resides.

The smooth muscle cells, which make up the intestinal and mononuclear tube as for the vessels, can contract and generate strength, and be electrically excited. There are smooth cells that determine a slow or tonic force of contraction and remain contracted for a prolonged period of time, and smooth cells with a faster or phasic contractile capacity whose action is expressed in a faster but shorter time [9]. Currently, we do not know the exact mechanisms that generate different forces and behaviours in smooth muscle cells [9]. Smooth muscle cells resemble striated ones, as they have many proteins and similar functions, such as actin, desmin, calponin, vinculin, integrin. They adapt to mechanotransduction stimuli, influencing the cell itself and the extracellular environment around it, towards other cells or tissues; they do not have organized protein bands (striated) [9]. Their organization resembles an analog system [9] (Figure 1).

The forces acting on the intestinal tube when food passes and stimulating mechanotransduction responses of smooth muscle cells are related to tangential transient forces generated on the internal mucosa (shear stress), and to forces linked to food pressure against the walls of the digestive tube (circumferential stretch). Shear stress mainly affects the more superficial smooth muscles, while the second one mainly concerns the outer cells. The smooth cells of the gastric system can expand their volume (temporarily or permanently) towards other smooth cells or other cellular structures, such as enteric or axonal ones, through interaction or incorporation; this mechanism allows them to gain faster information and faster adaptation [10]. Smooth cells can hypertrophy and undergo hyperplasia, depending on the mechanical and metabolic stimulus. Within the circular or longitudinal layers, there are other cells, such as fibroblasts, pericytes, telocytes and mast cells [10]. Smooth muscle cells are fundamental during development because they create an organization that allows the enteric cells to position themselves correctly and to maintain their position; the smooth cells allow the enteric system to function properly in the adult stage [11]. The possibility of emphasizing the contraction force produced towards the whole gastric tree (peristalsis) is given by the presence of some cells among the smooth cells: the interstitial cells. These cells form gap junctions between smooth muscle cells and are divided into two subclasses; the former is defined as c-kit+ interstitial cells of Cajal (also called telocytes), while the latter is known as platelet-derived growth factor receptor (APDGFRα+). The interstitial cells are found among muscular layers of the gastric tube [12].

Involuntary striated cells of the gastric tube

The gastrointestinal tract starts at the mouth and ends at the anus. The pharynx is a musculo-membranous tube leading from the oral and nasal cavities in the head to the oesophagus, the larynx, and the middle ear. The muscles of the pharynx are striated muscles divided into two muscular layers: the outer circular layer (superior constrictor muscle, middle constrictor muscle and inferior constrictor muscle) that constricts to propel bolus downwards, and the inner longitudinal layer (the salpingopharyngeus muscle, that opens the pharyngeal orifice of the pharyngotympanic tube during swallowing and raises the pharynx and larynx during deglutition, the stylopharyngeus muscle with the function of elevating the pharynx and larynx, and the palatopharyngeus muscle, that influences phonation) [13]. The pharynx is derived from the endoderm (pharyngeal pouch), but the pharyngeal muscles are derived from the visceral mesoderm [14]. These muscles are all striated muscles, with contractile proteins organized into sarcomeres, but they are involuntary and visceral muscles. The human oesophagus is composed of striated muscle proximally and of smooth muscle distally [15]. These different cells cooperate to distribute the peristaltic wave from the oesophagus to the stomach. The origin of the oesophagal striated cells is the lateral plate mesoderm [15].

Gallbladder and bile duct

The gallbladder has a single layer of smooth muscle tissue (muscularis propria), rich in smooth contractile cells, which derives from a mesodermal mesenchymal layer; through mechanical and chemical stimulation, this layer contracts to empty out the gallbladder [16]. Smooth muscle cells contain the c-kit + interstitial cells of Cajal [17]. The bile duct has only one muscular layer (muscularis mucosae), with limited contractile capacity, but with smooth muscle cells [17]. The origin of the muscular layer is always mesodermal. Smooth muscle cells in the presence of constant mechanical stress can choose to follow two strategies to avoid structural damage: reinforcement or fluidification. Through the first strategy, the most known, the cell polymerizes actin by increasing the assembly of the focal adhesions; this increases the cytoskeletal stiffness and the resistance that the cell has towards the same stressor [18]. With the second one, the smooth cell implements a fluidification mechanism, where it decreases the cytoskeletal stiffness, softening the cell; therefore, the cell offers less resistance to the existing forces, decreasing the risk of cellular damage [18]. The cell can choose whether to face the opposing force (reinforcement) or to follow its flow (fluidification). The result reflects the presence of the stressor temporally. Probably, the presence of ionic channels changes the behavior of smooth muscle cells in case of structural deformation, influencing the cellular response, such as calcium channels (L-type Ca2+), potassium channels, non-selective cation channels (for sodium, magnesium, calcium and potassium), chloride channels, sodium channels. These channels are found throughout the intestinal tract on smooth muscle cells [19].

Mesodermal development of the respiratory system

The larynx begins to develop during the first 10 weeks of gestation of the embryological period; the larynx, as does the entire respiratory system, originates from the laryngotracheal diverticulum, an evagination of the laryngotracheal groove or (shower) [20]. The laryngotracheal diverticulum (endoderm) is in front of the developing foregut. The diverticulum lengthens caudally (vertical slit) and is covered by a visceral mesoderm. The endoderm will originate the epithelium lining and the attached glands, while the mesoderm will generate the lamina propria, the cartilaginous structures and the vascular system [20]. The cartilages and laryngeal muscles derive from the IV and VI pharyngeal arch (mesoderm). The proliferation of the mesoderm in the cranial end of the diverticulum forms a pair of arytenoids, which transform the primitive glottis into laryngeal access by growing towards the tongue. The recesses will differ in vocal membranes and vestibular membranes (true and false vocal cords, respectively). The epiglottis develops from a proliferation of the mesenchyme of the third and fourth pharyngeal arch. The same proliferation forms the posterior one-third of the tongue. The muscles are innervated by the laryngeal ramifications of the vagus nerve [20]. The laryngotracheal diverticulum continues to grow to make space for trachea and lungs. The larynx is an organ of phonation, it assists the act of coughing and protects the trachea against food aspiration. The larynx is composed of three pairs of small cartilages (arytenoid, corniculate, cuneiform) and three large unpaired cartilages (thyroid, cricoid, epiglottis) but there are two synovial articulations or laryngeal joints: cricothyroid and cricoarytenoid [21]. The muscles of the larynx are divided into extrinsic muscles (above and below the hyoid bone) and intrinsic muscles, that are responsible for controlling sound production, as the lateral cricoarytenoid muscles and the posterior cricoarytenoid muscles [21]. We should consider the visceral joint, the visceral striated muscles and the laryngeal cartilages to define the fascia. The laryngotracheal diverticulum grows caudally covered by visceral mesoderm and it widens progressively to the terminal portion, where it forms a lung bud; the respiratory diverticulum also develops the trachea, while the respiratory bud splits into two extroflections to form primitive bronchial buds. These buds immediately form secondary and tertiary bronchial gems [22].

Trachea

The inner part of the trachea, the epithelium and the glandular structures have an endodermal origin; instead, its antero-lateral incomplete cartilaginous structure and the posterior part have a mesodermal origin; the posterior part joins the cartilage rings made of laryngeal muscles or smooth muscle fibres [22]. The posterior tracheal wall, that is in contact with the oesophagus and is behind it, adapts to the oesophagal peristalsis; in fact, the cartilaginous rings are incomplete to allow the trachea to collapse slightly so that food can pass down the oesophagus. During embryological development, the mesoderm influences the internal conformation of the trachea through some growth factors, such as fibroblast growth factor-10 (FGF-10), epidermal growth factor (EGF), insulin-like growth factor-1 (IGF-1), stimulating the endoderm. In a fully developed trachea, the mesoderm maintains its shape, through the cartilage rings, and its function, through smooth muscle contraction [22]. The tracheal epithelium, that forms the internal mucous membrane, is formed by basal and ciliated cells-club and globet secretory; the trachea in its proximal portion has a larger number of basal cells [23]. The smooth muscle cells allow the movement of epithelial cells. Muscle cells produce oscillatory contraction waves steadily (like peristalsis). The activation of L-type voltage-dependent calcium channels (LVDCCs) allows Ca2+ to enter the cytosol; this increase in calcium will activate the opening of Clca channels, which will mediate chloride efflux and membrane depolarization, also activating LVDCCs for an optimal contraction. The calcium concentration in the cytosol is managed by Na+-Ca2+ exchanger (NCX), that places one calcium molecule in the extracellular space and three sodium molecules in the cytoplasm, inducing a decreased amount of calcium and muscles relaxation [24]. Rhythmic contraction is supported by the deformation of the contractile smooth cell [24]. Cytoplasmic calcium activates Ca2+/calmodulin-dependent Myosin light-chain kinase (MYLC or MLCK) that phosphorylates a specific myosin light chain (RLC) associated with the actin to produce contractile strength. A decrease in calcium activates the Myosin-light-chain phosphatase (MLCP) enzyme that dephosphorylates RLC, producing muscle relaxation. The smooth muscle behind the trachea and the cartilage (the inner layer) does not have interstitial cells, contrary to gastrointestinal smooth muscle cells [25]. Potassium channels activity (Kv7.5) is another mechanism that reduces intracellular calcium and allows muscle relaxation, through the hyperpolarization of the cell membrane and the mechanism allowing sodium and calcium to enter the cell and potassium to exit the cell [26]. The trachea has the function to humidify incoming air and to expel substances, organisms and particles from the immune and respiratory system by cyclic movements of the epithelium smooth muscle; the seromucous gland is interposed in the connective tissue and is influenced by the mechanotransduction stimuli of the smooth muscle cells [22]. The innervation of the trachea is derived from branches of the recurrent vagus nerve and the anterior pulmonary plexus.

Bronchi

The respiratory bud forms the primitive bronchial buds, dividing into two extroflexions during the fifth week of pregnancy. These immediately form secondary and tertiary bronchial buds [22]. A bronchus is a passage of airway in the respiratory system that conducts air into the lungs. The first bronchi to branch from the trachea are the right main bronchus and the left main bronchus (carina of the trachea). These two bronchi have the same structure and they enter the lungs at each hilum, where they branch into narrower secondary bronchi, and these branch into narrower tertiary bronchi, forming the respiratory tree. While the two main bronchi and part of the secondary bronchi are extrapulmonary bronchi, after entering the lungs the bronchi become the intrapulmonary bronchi. The respiratory tree includes the bronchi and bronchioles and it ends in the lung parenchyma as terminal bronchioles. The outer cartilaginous and muscular layer derives from the mesoderm, while the innermost and middle layer (ciliary and mucous) derives from the endodermal sheet. The bronchi are innervated by the sympathetic and parasympathetic plexus (tonic activity in smooth muscles); the neurotransmitters are the catecholamines for β receptors in the sympathetic system and the acetylcholine in the parasympathetic system [22].

Lungs

Endoderm and mesoderm contribute to lung development. The pulmonary epithelium derives from the ventral endoderm, while the pulmonary parenchyma origins from the mesoderm; the parietal pleura will result from the lateral mesoderm, while the visceral pleura will result from the visceral mesoderm [27-28]. In the process of lung development, we can identify five phases: embryological phase; pseudoglandular phase; canalicular phase; saccular phase; alveolar phase. The embryological phase includes (four to seven weeks), during which the pulmonary buds appear from the endodermal primitive gut tube; the lobular structures of the future lungs begin to form during this stage [29]. In the second phase (seven to 15 weeks), the lungs continue to be branched, and structures such as cartilage, smooth muscle cells, mucous glands and the epithelium begin to form [29]. The first fetal breath appears between the 10th and 11th week. During the canalicular phase (16 to 26 weeks), the lungs prepare the space for the alveoli and the lung volume increases. During the saccular phase (24 to 38 weeks), the pulmonary arborization ends and the differentiation of alveolar epithelial cells continues. The alveolar phase is the process of alveolar formation, from 36 weeks to three years of age, and according to some authors, this growth persists up to 21 years [29]. Before birth, the pulmonary epithelium has many chloride channels for the amniotic fluid; after birth, a decrease of chloride channels occurs due to an increase in sodium channels to absorb water, stimulated by beta-adrenergic signals [29]. The mesoderm gives multiple biochemical and structural information to the lungs [27]. From the mesoderm derives the fibroblast growth factor-10 (FGF-10) (essential for growth and survival), bone morphogenetic protein-4 (Bmp4) (for growth of pulmonary epithelium), and other substances needed for the differentiation of the lung mesoderm (sonic hedgehog homolog or Shh, Wingless-related integration site or Wnts, vascular endothelial growth factor or VEGF, platelet-derived growth factor or PDGF, transforming growth factor beta or TGF-β), such as pericytes, parabronchial smooth muscle cells, and myofibroblasts [30]. The mechanical movement of the formed lungs allows to stimulate the tissue derived from the mesoderm and to adapt to the environment for maintaining their shape and function [27]. For example, sensory receptors in the visceral pleura mediate mechanical and pain signals towards the central nervous system; this mechanism allows the visceral pleura to adapt and implement responses to deal with possible pathogens. Furthermore, this pleural receptor capacity allows mechanical information to be transmitted to the whole lung so that the respiratory acts are more effective [28]. Mechanical stimulation of the parenchymal and pleural tissue can also stimulate ion channels (sodium and potassium), which will stimulate smooth muscle cell responses [31]. The innervation of the lungs is complex due to the intervention of somatic fibres (intercostal nerves), sympathetic and parasympathetic fibres [28].

Spleen

The development of the spleen starts around the fifth week of gestation, deriving from the visceral mesoderm. Until the fifth month of gestation, when the bone marrow begins to function, the spleen has important hematopoietic functions. After birth, no significant hematopoietic function remains (except in pathological conditions) [32]. The spleen is a large lymphoid organ located in the abdominal cavity, on the left, beneath the diaphragm. It is surrounded by a thick connective capsule (parenchyma) with numerous connective tissue septa, which penetrate the spleen without dividing it into lobes but forming a fine reticular texture that supports the parenchyma. The parenchyma of the spleen contains two main types of tissue, white pulp and red pulp, separated by the marginal zone that is a highly transited area that receives large amounts of blood from the general circulation; furthermore, within the marginal zone there exist two types of macrophages that are unique to this area. The red pulp filters the blood of antigens, microorganisms, and defective or worn-out red blood cells; the white pulp activates the immune response in the presence of antigens [32]. The innervation follows the vascular system in the hilum of the spleen and inside it, the vagal branches always remain in contact with the blood vessels [33]. The spleen is rich in fibroblasts and smooth muscle cells with alpha-actin (and other proteins, such as cytokeratins, and laminins): these structures constitute the parenchyma [34]. The presence of contractile proteins is not enough to change the mechanical or morphological properties of the parenchyma. The mechanisms of mechanotransduction in the spleen during diaphragm movement, the resistance which comes from other organs or the adaptive splenic ability to vary the blood and lymphatic shear stress, is not yet known.

Peritoneum

The peritoneum is the largest of the serous membranes, with a surface area of 1.8 m^2^. The peritoneum is a thin, transparent serous membrane that consists of two layers: it forms the lining of the abdominal cavity and part of the pelvic cavity (parietal peritoneum) and it covers the surfaces of most of the contained viscera (visceral peritoneum), fixing them to the abdominal wall through mesenteries and ligaments. The peritoneum formation begins in the gastrulation stage with the coelom formation. The peritoneal cavity derived from the caudal end of the coelomic cavity, while the peritoneal membrane derived from the mesoderm (between the 25th and 28th day). The parietal peritoneum is derived from the parietal mesoderm, while the visceral peritoneum is derived from the visceral mesoderm; an exception is the visceral peritoneum of the liver, which is derived from the parietal mesoderm. The peritoneum gives rise to many connected structures, as visceral ligaments, the mesentery, Toldt's fascia, greater omentum and lesser omentum, and it is in contact with different abdominal and pelvic viscera, always remaining a unique structure. The peritoneum consists of three layers: the mesothelium, the lamina basal and the submesothelial stromal layer. The innermost layer of the peritoneum, the mesothelium, is a single layer of mesothelial cells, with epithelial and mesenchymal properties; the cellular morphology depends on the properties of the viscera (flattened, cuboidal and mixed). Mesothelial cells are connected by intercellular junctions (gap junctions, tight junctions, desmosomes), and they can differentiate into adipocytes in case of metabolic needs. The lamina basal supports the previous layer and it is composed of extracellular matrix (type IV collagen, laminin). The submesothelial stromal layer is rich in elastic cellular structures that form an elastic lamina, such as type I collagen, laminin, fibronectins, glycosaminoglycans, fibroblasts, proteoglycans, fat cells, blood and lymphatic vessels and neural pathways. This layer is also known as the interstice. The submesothelial stroma is in connection with the intestinal serosa (outermost layer) in the mesentery: the connective tissue of the mesentery is in continuity with the elastic tissue of the intestine [35]. The mesentery is a contiguous set of tissues that attaches the intestines (jejunum and ileum) to the posterior abdominal wall and is formed by the double fold of peritoneum; the mesentery derives from the lateral plate of the mesoderm [8]. The parietal peritoneum is innervated by the spinal nerves: cranially by the phrenic nerve and caudally by the thoracoabdominal nerves and the lumbosacral plexus. The obturator nerve innervates the parietal peritoneal portion of the pelvis [35]. The receptors in the parietal peritoneum are pressure sensitive (important for mechanotransduction) and heat sensitive, as well as nociceptive receptors. The innervation of the visceral peritoneum is complex and concerns the celiac and mesenteric sympathetic plexus, as well as the parasympathetic system: the receptors are sensitive to stretch and to chemical stimuli (mechanotransduction) [35].

Urogenital system

The genitourinary system develops from the intermediate mesoderm or nephrogenic cord, except for the bladder and the urethra epithelium which have an endodermal origin. The nephrogenic cords are bilateral mesenchymal densities that extend from the cervical to the sacral tract. These cords take on a metameric aspect in the cervical tract and in the first thoracic tract (nephrotomes). The cervical nephrotomata that develop first give rise to the pronephros, and the upper thoracic and lumbar ones subsequently evolve in the mesonephros and the remaining lumbar and sacral blastema forms the metanephros. The pronephros disappears after one day (between 24 and 25 days), to be replaced by the mesonephros; the pronephros generates, before disappearing, an epithelial duct or nephritic duct or pronephric excretory duct [36]. The proximal tract of this duct disappears, while the distal portion connects to the duct/tubule of the mesonephros. The medial part of each mesonephric tubule connects with capillaries to form Bowman's capsule, constituting a filtration unit known as the renal corpuscle; the lateral part of each mesonephric tubule continues as a mesonephric duct or Wolffian duct [36]. The mesonephros regresses to give space to the metanephros; the latter originates from two distinct buds. Thus, we have the ureteric gem and the metanephrogenic blastema or nephrogenic mesoderm, which will stimulate a reciprocal differentiation generating the collector and the excretory system. The collector system originates from a diverticulum of the Wolffian duct, the ureteric bud (near the cloaca) surrounded by metanephric tissue; the proximal part of this diverticulum represents the future ureter while the dilated portion repeatedly divides and forms the pelvis, the renal calyces, the channels and the collecting tubules of the definitive kidney [36]. The metanephrogen blastema, the last part of the nephrogenic cord, will form the future excretory system. The neurotrophic factor derived from glial cells (GDNF) represents the primary branching stimulator of the ureteric bud, while fibroblast growth factor-8 (FGF-8) allows the branching and lengthening of the ureteric bud. Other growth factors are the Wingless-related integration site-8 (Wnt-8) (for the sequential formation of the pronephros and mesonephros) and the bone morphogenetic protein (BMP) (important for the branching and lengthening of the ureteric bud) [36-37]. Kidneys development is based on a strong mechanical component (mechanotransduction). The direction of the cells that will form the definitive kidney is caudal; continuing to form cells (mesonephros), the latter will undergo an elongation. The cells can duplicate themselves, through Wnt-8, to be resistant to mechanical stretch tension [37]. The mesoderm then will stimulate the endoderm, via FGF-2, to build the epithelial layer of kidneys and ureter [38]. The final kidney forms initially in the pelvic region and then rises into the lumbar region; this displacement will cause the lengthening of the ureter, rich in smooth muscle cells [36]. The finished kidney is covered by a fibrous capsule, a thin connective tissue covered with fat and the renal fascia; below the fibrous capsule, there is the renal parenchyma, separated from the muscle tissue of the kidney. In these three renal layers, there are many connective cells, fibroblasts and smooth muscle cells capable of contracting, telocytes and podocytes, as well as multiple vessels and related connective cells (pericytes) [39]. Below the parenchyma, we find the cortical area and finally the medullary area [39]. Podocytes are cells capable of contracting, with low levels of alpha-actin; they are found in the glomeruli and in the corpuscles [38]. The blood vessels possess many cells capable of contracting, as well as the parietal cells of the Bowman capsule (cells with smooth muscle myosin) [39]. The cells and the structure of the kidney adapt to liquid stimuli (shear stress) and from mechanical stimuli originating in other tissues (diaphragm, viscera connected to kidneys). We still know very little about the physiology of the mechanotransduction capacity of the renal structures. The kidney is innervated by the sympathetic system [38].

Adrenal gland

The adrenal gland begins to develop from the urogenital field, a condensation of celomic mesodermal cells, between the third and fourth week of gestation; the neural crest cells (ectoderm) will infiltrate the gland in transformation, which will form the medullary portion of the gland [40]. Towards the ninth week of gestation, the glandular primordium is completely enveloped by what will become the glandular capsule (connective tissue) [40]. In the gland, we can find three zones: the external glomerular area (for the synthesis of mineralocorticoids), the intermediate fasciculata zone (for the synthesis of glucocorticoids) and the most internal reticular zone (for the synthesis of androgens) [40]. The gland is innervated by the sympathetic celiac plexus. The glands are closed by the renal fat and the renal fascia [41]. The muscles of the head and neck have a double derivation, exactly as the adrenal gland: mesodermal and ectodermal origin [5]. It, therefore, can be considered fascia. We know little about the normal behaviour of the gland in front of mechanical stimuli.

Urinary bladder

The urinary bladder is responsible for collecting the urine produced by the kidneys and transported by the ureters. The bladder derives from the endoderm, for the epithelial lining (urothelium), and from the mesoderm for the muscles (smooth muscle cells and fibroblasts) [42]. Muscle cells are the first to appear (seven to 10 weeks of gestation). At the end of the second trimester, the bladder loses its peristaltic conduit shape to become a container with small unordered movements; a circular musculature develops at the neck of the future bladder [42]. At the 15th week of gestation, the urethra presents involuntary striated muscles. The bladder is formed from the cranial portion of the primitive urogenital sinus, separated from the cloaca thanks to the urorectal septum. The primitive urogenital sinus expands by incorporating the terminal segments of the Wolffian duct (mesoderm) and ureters into the posterior part. The orifices will change position: those of the ureters pass up and to the side, while those of Wolff end up dragged down. These movements end up leaving part of the mesonephric tissues incorporated in the posterior wall of the bladder, forming a triangular-shaped structure that takes the name of trigon (triangular area whose posterior angles are constituted by the two ureteral orifices and the anterior angle from the internal urethral orifice) [43]. At the end of the third month, the allantois obliterates forming the cord that will suspend the bladder to the anterior wall of the abdomen, the urachus [43]. The bladder wall consists of different tissues: the internal mucous tunic (epithelium of lining), proper connective tunic or lamina propria, the tunica muscularis (smooth musculature, contains the detrusor muscle of the bladder), subdivided into three layers (inner or plexiform layer, middle or circular layer, outer or longitudinal layer); tunica adventitia (outer layer); serous membrane (formed by the peritoneum at the apex and in some parts of the bladder) [43]. The fundamental growth factors for correct bladder development and balanced growth between mesoderm and endoderm are different: SHH, bmp4, TGF-beta, fibroblast growth factor receptor 2 or FGFR2 [43]. The innervation of the bladder is very complex. Various structures intervene, such as the sacral parasympathetic nerves, the hypogastric sympathetic chain and the pudendal nerves [42].

Genital organs

The male and female gonads originate from the gonadal or genital crests; these consist of mesenchyme and cells of mesonephric origin. The gonads derive from the intermediate mesoderm [7]. The gonadal crest appears after about five weeks and gives rise to the sexual cords. In the sixth week, the genital crests are invaded by primordial germ cells (endodermal derivation from the yolk sac). Thanks to this cellular migration, the gonads begin to differentiate (males and females). In the male, some mesonephros tubules will participate in the formation of the genital system, while in the females these tubules will disappear [44]. The male sexual cord penetrates the medulla gonadal, forming the medullary cords of the testis; in testis cords, we will find the sustentacular cells of Sertoli and the interstitial Leydig cells. The mesonephros will form the ductus deferens and the ductuli efferentes [44]. In the female the sexual cord will form groups of irregular cells, which will occupy the medullary part of the ovary, developing the organization of the ovary. The presence of mesoderm is essential to maintain the correct sexual differentiation: testicles and ovaries [44]. The finished testicle is covered by the tunica vaginalis with a peritoneal origin (mesoderm); the testicle is constituted by the albuginea tunic, the parenchyma (with semiform and rectal tubules) and the stroma [45]. The testicles are rich in connective tissue, fibroblasts and smooth muscle fibres, as well as spermatic ducts [45]. The ovary is covered by the epithelium in continuation with the peritoneal mesothelium; below, there is a connective layer (false albuginea), the parenchyma, the cortical (fibroblasts, collagen fibres) and, deeply, the medullary area. The innervation of the testicle derives from the celiac plexus of the orthosympathetic which reaches the testicle following the blood vessels and forming a rich testicular plexus which also receives a parasympathetic component from the deferential plexus. The ovarian innervation comes through the arterial vessels around which they form the uterus-ovarian plexus, part of the celiac plexus; these are adrenergic and cholinergic myelinated fibres, mostly destined for vasomotor innervation [45].

Uterus

At about the eighth week, the uterus, fallopian tubes and upper third of the vaginal canal develop from the mesonephric duct, thanks to the absence of Mullerian inhibiting factor, that is present in males [46]. We can find three layers in the uterus: the endometrium (the internal mucosa), the myometrium (the muscular layer with smooth muscle cells), the most external or serosa/perimetrium layer. The somatic innervation derives from T11/T12, the parasympathetic innervation comes from S2/S4, while the sympathetic innervation derives from the hypogastric plexus [46].

## Conclusions

Tissues consist of smooth muscle and non-voluntary striated musculature, originated from the mesoderm and with the properties described, they have never been properly considered. They are some of the viscera present in the mediastinum, in the abdomen and in the pelvic floor. The article highlights the presence of smooth and visceral striated muscle cells in different organs, underlying the mesodermal origin. Mesoderm during organogenesis is essential for giving shape and function to organs. Osteopathic clinical practice and teaching osteopathic medicine include the visceral manual approach. Knowing that some viscera satisfy the definition of what fascial tissue is, will allow the osteopath to improve its practice. In the second part of the article, we will give a conclusive definition of fascia and we will explain the embryological development of the heart and we will see how the fascial tissue can be subject to manual treatment.
